# Prevalence and factors associated with inadequate intake of fruits and vegetables in a population from Northern Angola

**DOI:** 10.4314/ahs.v25i1.32

**Published:** 2025-03

**Authors:** Humberto Morais, Vizé Preciosa Cupessala, João Mário Pedro, Miguel Brito, Mauer AA Gonçalves

**Affiliations:** 1 Centro de Estudos Avançados em Educação e Formação Médica, Faculdade de Medicina, Universidade Agostinho Neto, Luanda, Angola; 2 Hospital Militar Principal/Instituto Superior, Luanda, Angola; 3 Instituto Gulbenkian de Ciência, Oeiras, Portugal; 4 Centro de Investigação em Saúde de Angola (CISA), Angola; 5 Health and Technology Research Center (H&TRC), Escola Superior de Tecnologia da Saúde de Lisboa, Instituto Politécnico de Lisboa, Portugal

**Keywords:** Fruits and vegetables, non-communicable chronic diseases, sociodemographic factors, Angola

## Abstract

**Background:**

The World Health Organization recommends a minimum intake of 400 g or five servings of Fruits and Vegetables (FVs) per day for the prevention of chronic diseases.

**Objective:**

The present study aims to describe the prevalence and factors associated with inadequate FVs intake in a sample of Angolan adults who participated in the CardioBengo study.

**Methods:**

It is a subset analysis of CardioBengo, a community-based cross-sectional observational study conducted in the Dande Municipality, Bengo Province, Angola.

**Results:**

The sample included 2161 individuals aged 18 to 84, with 64.1% being women. 57.2% of participants was below high school, and only 3.1% attended higher education. 61.7% were married, 48.3% had a monthly income below 150 USD. The prevalence of insufficient FVs consumption in the sample was 86.2%. It was observed that female gender, low education level, and the age group of 20-29 years were associated with inadequate FVs intake (p = 0.010, p = 0.001, and p = 0.006, respectively).

**Conclusion:**

There was no association between FVs consumption and cardiovascular risk factors. A prevalence of FVs consumption well below current recommendations was identified. The identified risk factors can serve as a strategy to increase FVs consumption in this population.

## Introduction

Worldwide, low intake of fruits and vegetables (FVs) significantly contributes to the burden of diseases, including cardiovascular diseases and cancer^1^. The World Health Organization (WHO) recommends a minimum intake of 400 g or five servings of FVs per day for the prevention of chronic diseases such as heart disease, cancer, diabetes, and obesity^2^. A study involving 52 low- and middle-income countries revealed that, among an adult population (18-99 years), the global prevalence of inadequate FV intake was 77.6% for men and 78.4% for women^3^, with wide variations between countries (from 36.6% and 38.0% in Ghana to 99.2% and 99.3% in Pakistan). In Asia, inadequate FV intake exceeded 89% in men and 96% in women in Bangladesh; 96% in men and 92% in women in Indonesia, and over 84% in men and over 79% in women in Thailand^4^. On the other hand, a study among the adult population in South Africa, published in 2004, found that 72.2% of men and 66.7% of women had inadequate FV intake^5^. In a similar vein, another study, also conducted in South Africa but among the elderly, showed that the proportion of inadequate FV consumption was 64.8% in men and 71.4% in women^6^. In contrast, in Mozambique, less than 5% of adults (25-64 years) reported a daily intake of five or more servings of FVs^7^. Recent studies in Sub-Saharan Africa also demonstrated insufficient FV intake among adults. Msambichaka et al. in Tanzania^8^, Pengpid et al. in Kenya^9^, and Yaya et al. in Namibia^10^ reported prevalence rates of inadequate FV intake at 83%, 94.8%, and 95.8% in men, and 82%, 93.2%, and 95.2% in women, respectively. Several factors have been identified as associated with the prevalence of inadequate (insufficient) FV intake, including: a) Sociodemographic data: young age^11^, male^12^ or female^9^,^13^ gender, lower education^8^, lower income^9^, unmarried status^12^,^14^ b.) Chronic diseases and other conditions such as: diabetes mellitus^15^, hypertension^16^, overweight or obesity^17^, hypercholesterolemia^18^,^19^ c.) Lifestyle factors: smoking^9^, sedentary behavior^20^,^21^, alcohol consumption^8^. Data on fruit and vegetable consumption are scarce in Angola; therefore, the present study aims to describe the prevalence and factors associated with inadequate FV intake in a sample of Angolan adults who participated in the CardioBengo study.

## Methods

### Study Type and Location

The present research is a subset analysis of CardioBengo, an analytical cross-sectional observational study conducted in the catchment area of the Dande-Health Demographic Surveillance System (Dande-HDSS), located in the Dande Municipality, Bengo Province, Angola^22^. This research, conducted between September 2013 and March 2014, serves as a broader baseline on cardiovascular risk factors^23^, using the methodology proposed by the World Health Organization (WHO) STEP-wise Approach to Surveillance (STEPS) to Chronic Disease Risk Factor (Basic and Expanded version 3.0) manual^24^.

### Study Population

The sample size included 2161 individuals aged 18 to 84, divided into six age groups ranging from 18 to 84 years.

### Data Collection

Participants were assessed by trained interviewers and certified healthcare professionals. As described in the CardioBengo study protocol^23^, information on sociodemographic characteristics, tobacco consumption, and dietary habits was collected through an interview following the STEPS manual. Blood pressure and all clinical measurements were obtained using point-of-care devices, namely, OMRON M6 Comfort automatic sphygmomanometer (OMRON Healthcare Europe BV, Hoofddorp, Netherlands); ACCU-CHEK Aviva blood glucose meter (Roche Diagnostic, Indianapolis, USA); and ACCUTRENPlus (Roche Diagnostic, Indianapolis, USA) with ACCUTREND CHOLESTEROL test strips (Roche Diagnostic, Indianapolis, USA). Waist and hip circumferences were measured with an accuracy of 0.1 cm using a SECA 203 circumference tape (SECA UK, Birmingham, UK), body weight measured to the nearest 0.1 kg using a SECA 803 digital scale (SECA UK, Birmingham, UK), height measured with an accuracy of 0.1 cm in a standing position using a portable SECA 213 stadiometer (SECA UK, Birmingham, UK).

### Variables under Study

This study included sociodemographic variables: age, age group, gender, education level, and family income; Behavioral variables: smoking, alcohol consumption, fruit and vegetable intake; Clinical variables: blood pressure, blood glucose, total cholesterol, body mass index (BMI), abdominal obesity. For analysis, age was categorized into the following groups: less than 20 years, 20-29, 30-39, 40-49, 50-59, and 60 years or older. Education was classified based on the number of completed study years as none, basic education 1 to 6 years; middle school I cycle 7 to 9 years; middle school II cycle 10 to 12 years; and higher education. Marital status was categorized into three categories: single, divorced, widowed (living alone); single (living with parents); married (living with a partner). Monthly family income in kwanzas was converted to US dollars (USD) at the 2014 exchange rate and categorized into groups: no income; less than or equal to 150 USD; 151 to 299 USD; and greater than or equal to 300 USD. BMI and waist-to-hip ratio were calculated and categorized according to WHO guidelines. BMI was categorized as underweight (<18.5 kg/m^2^), normal (18.5 to 24.99 kg/m2), overweight (25.0 to 29.99 kg/m2), and obese (≥30 kg/m2). Abdominal fat was considered if the waist-to-hip ratio was greater than 0.90 in men and greater than 0.85 in women^25^. Fruit and vegetable intake was categorized as low intake if less than five servings of FVs per day, less than 5 days per week, and adequate intake if more than five servings of FVs per day, on five or more days per week. Hypertension was defined when systolic blood pressure was higher than 140 and/or diastolic blood pressure was 90 mmHg. Hyperglycemia was considered when fasting blood glucose was equal to or higher than 126 mg/dl, or casual blood glucose was equal to or higher than 180 mg/dl. Hypercholesterolemia was considered when total cholesterol was equal to or higher than 200 mg/dl. Smoking was categorized as never smoked, ex-smoker, and current smoker. Alcohol consumption was categorized as never drank, ex-drinker, and current drinker.

### Inclusion and Exclusion Criteria

Residents in the Dande-HDSS study area aged 18 years or older were included in the study. Individuals with unrecorded anthropometric or blood pressure values were excluded. Pregnant individuals were also excluded from the study due to changes in their biochemical and anthropometric parameters inherent to pregnancy.

## Ethical Considerations

All procedures conducted in this study were approved by the Ethics Committee of the Ministry of Health of Angola, in accordance with its guidelines and the Helsinki Declaration of 1964 and its subsequent amendments. Written informed consent was obtained from all participants.

## Statistical Analysis

Data were entered into Microsoft Excel 2017 and imported into the Statistical Package for the Social Sciences (SPSS®) version 26.0 (IBM, New York, USA) for data analysis. Descriptive analysis was performed by calculating measures of central tendency, such as mean, median, mode, and standard deviation for quantitative variables, and frequencies and percentages for categorical variables. Analytical testing was conducted using Chi-square (χ2) and Mann-Whitney tests. A confidence interval of 95% (CI 95%) was considered for all calculated proportions.

## Results

### Sample Characteristics and FV Consumption Rate

The total sample included 2161 individuals aged 18 years or older, with an average age of 36.93±13.86 years, and 64.1% were women. The majority of participants (57.2%) had an education level below high school, and only 3.1% had or were attending higher education. A large majority were married (61.7%). Of those who reported family income, 28.9% had a monthly income below 150 USD. From the total sample, 21.0% were hypertensive, 17.6% had hypercholesterolemia, 10.4% had hyperglycemia, 8.7% were obese, and 32.3% had abdominal fat. About 38.8% were alcohol users, while a small portion of participants were smokers (6.8%). The prevalence of insufficient consumption in the total population of FVs was 86.2% (65.2% in women and 34.8% in men). The average intake per day was 3.3 servings for FVs (1.7 for fruits and 1.6 for vegetables) (Table 1).

**Table 1:** Descriptive statistics of sample characteristics and prevalence of insufficient fruit and vegetable consumption

	Total sample	Men	Women	Total	Fruits	Vegetables	Fruits and Vegetables
	
	N (%)	Inadequate Fruit and Vegetable Consumption in %				Average Servings/Day (DP)	
Total	2161(100.0)	83.6	87.6	86.2	1.7(1.5)	1.6(0.8)	3.3(1.7)
**Age Class**							
<20	232(10.7)	91.2	90.7	90.9	1.9(1.4)	1.3(0.7)	3.2(1.8)
20 – 30	621(28.7)	84.1	85.4	84.9	2.0(1.6)	1.5((0.8)	3.5(1.8)
30 – 39	441(20.4)	76.2	86.7	83.2	1.8(1.3)	1.7(0.8)	3.5(1.6)
40 – 49	358(16.6)	75.3	87.5	84.4	1.7(1.4)	1.6(0.8)	3.3(1.7)
50 – 59	366(16.9)	86.5	88,5	88.0	1.5(1.4)	1.7(0.8)	3.2(1.7)
>60	143(6.6)	94.3	92.2	93.0	1.2(1.2)	1.6(0.8)	2.9(1,5)
**Level of Education**							
None n(%)	313(14.5)	100.0	93.3	92,7	1.3(1.4)	1.7(0.8)	3.0(1.6)
Basic Education n(%)	922(42.7)	83.0	87.7	86.7	1.6(1.3)	1.6(0.8)	3.2(1.6)
Secundary Education 1st cycle n(%)	444(20.5)	83.7	84.4	84.0	2.0(1.4)	1.4(0.8)	3.5(1.8)
Secondary Education 2^nd^ cycle n(%)	415(19.2)	83.9	83.9	83.9	2.1(1.7)	1.5((0.9)	3.6(2.0)
Higher education n(%)	67(3.1)	79.0	69.2	77.6	1.9(1.5)	1.7(0.9)	3.6(1.9)
**Family Income**							
None n(%)	126(5.8)	84.0	91.1	89.3	1.4(1.2)	1.7(0.7)	3.1(1.5)
≤150 USDn(%)	625(28.9)	86.9	87.3	87.2	1.7(1.4)	1.6(0.8)	3.3(1.6)
151-299 n(%)	375(17.4)	77.1	88.1	84.0	1.8(1.5)	1.6(0.8)	3.3(1.8)
≥300 USD n(%)	167(7.7)	80.2	82.1	80.8	2.0(1.6)	1.7(0.9)	3.7(1.9)
**Marital status**							
Lives alone n(%)	342(15.8)	88.5	89.8	89.5	1.5(1.5)	1.6(0.8)	3.1(1.8)
Married n(%)	1333(61.7)	80.3	87.4	85.0	1.7(1.4)	1.6(0.8)	3.4(1.7)
Lives with parents n(%)	458(21.2)	88.7	85.5	87.1	1.9(1.6)	1.4(0.8)	3.3(1,9)
**FRCV**							
HTA	453(21.0)	86.4	87.6	87.2	1.5(1.3)	1.7(0.8)	3.2(1.6)
Hyperglycaemia	225(10.4)	84.9	89.2	87.6	1.5(1.2)	1.6(0.8)	3.1(1.5)
Hypercholesterolemia	380(17.6)	84.8	87.0	86.6	1.5(1.4)	1.6(0.8)	3.2(1.7)
Obesity	188(8.7)	81.5	87.0	86.2	1.4(1.3)	1.7(0.8)	3.3(1.7)
Abdominal Fat	699(32.2)	79.7	87.8	86.4	1.6(1.4)	1.7(0.8)	3.3(1.7)
Smoking Habits	146(6.8)	87.6	91.8	89.0	1.4(1.6)	1.6(0.8)	3.1(1.8)
Alcoholic Habits	839(38.8)	84.4	86.1	85.3	1.8(1.5)	1.6(0.8)	3.4(1.7)

### Predictors of Insufficient FLV Consumption

Female individuals, low education levels, and specific age groups were associated with inadequate FV intake (Tables 2 and 3). A significantly higher proportion of women had inadequate FV intake (68.2 vs. 34.8%, p = 0.010) when compared to the opposite gender. Similarly, participants with no education had a significantly higher but elevated proportion of inadequate FV intake compared to other education levels (p = 0.001). Regarding age groups, individuals under 20 years and those over 50 had a higher proportion of inadequate FV intake compared to other age groups (p = 0.006).

**Table 2:** Sociodemographic characteristics in the total population and according to adequate vs. inadequate consumption of fruits and vegetables

	Classification of Fruit and Vegetable Intake			
	Total N=2161	Sufficient N = 299	Insufficient N = 1862	*p*-value
Age (years) M±DP	36.93±13.86	37.05±14.08	36.06±12.35	.566
**Age Class**				.006
<20 n(%)	232(10.7)	21(7.0)	211(11.3)	
20 - 30 n(%)	621(28.7)	94(31.4)	527(28.3)	
30 - 39 n(%)	441(20.4)	74(24.7)	367(19.7)	
40 - 49 n(%)	358(16.6)	56(18.7)	302(16.2)	
50 - 59 n(%)	366(16.9)	44(14.7)	322(17.3)	
>60 n(%)	143(6.6)	10(3.3)	133(7.1)	
**Sexo**				.010
Masculino n(%)	775(35.9)	127(42.5)	648(34.8)	
Feminino n(%)	1386(64.1)	172(57.5)	1214(65.2)	
Level of Education				.001
None n(%)	313(14.5)	23(7.7)	290(15.6)	
Basic Education n(%)	922(42.7)	123(41.1)	799(42.8)	
Secundary Education 1st cycle n(%)	444(20.5)	71(23.7)	373(20.0)	
Secundary Education 2nd cycle n(%)	415(19.2)	67(22.4)	348(18.7)	
Higher education n(%)	67(3.1)	15(5.6)	52(2.8)	
Family Income				.074
None n(%)	126(5.8)	13(4.7)	113(6.1)	
≤150 USD n(%)	625(28.9)	80(26.9)	545(29.3)	
151-299 n(%)	375(17.4)	60(20.1)	315(16.9)	
≥300 USD n(%)	167(7.7)	32(10.7)	135(7.3)	
Marital status				.081
Lives alone n(%)	342(15.8)	36(12.0)	306(6.1)	
Married n(%)	1333(61.7)	200(66.9)	1133(60.8)	
Lives with parents n(%)	458(21.2)	59(19.7)	399(21.4)	

**Table 3:** Clinical and behavioral characteristics in the total population and according to adequate vs. inadequate fruit and vegetable intake

	Classification of Fruit and Vegetable			
	Total N=2161	Sufficient N = 299	Insufficient N = 1862	*p*-value
BMI M±DP	23.29±4.55	23-63±4.68	23.23±4.54	.099
**BMI**				.252
Low weight	172(8.0)	20(6.7)	152(8.2)	
Normal	1363(63.1)	181(60.5)	1182(63.5)	
Overweight	430(19.9)	72(24.1)	358(19.2)	
Obesity	188(8.7)	26(8.7)	162(8.7)	
**Abdominal Fat**				.760
Yes	699(32.2)	95(31.8)	60632.4)	
No	1449(67.1)	204(68.2)	1245(66.9)	
**Fruit and vegetable intake**				
Fruit servings/day Average ±DP	1.75±1.47	3.78±1.56	1.42±1.15	<.001
Vegetables servings/day Average ±DP	1.58±0.82	2.26±0.91	1.47±0.75	<.001
Fruit & Vegetable Servings/day Average ±DP	3.33±1.73	6.15±1.41	2.89±1.34	<.001
**Smoking Habits**				.600
Never smoked	1828(84.6)	255(85.3)	1573(84.5)	
Ex-smoker	174(8.1)	24(8.0)	150(8.1)	
Smoker	146(6.8)	16(5.4)	130(7.0)	
**Alcoholic habits**				.216
Never drank	925(42.8)	115(38.5)	810(43.5)	
Former Drinker	390 (18.8)	61(20.4)	329(17.7)	
Drinker	839(38.8)	123(41.1)	716(38.5)	
**HTA**				.488
Yes	453(21.0)	58(19.4)	395(21.2)	
No	1706(78.6)	270(80.3)	1661(78.7)	
**Hyperglycaemia**				.527
Yes	225(10.4)	28(9.4)	197(10.1)	
No	1931(89.4)	270(90.3)	1661(89.2)	
**Hypercholesterolemia**				.599
Yes	380(17.6)	51(17.1)	329(17.7)	
No	1311(60.7)	190(63.5)	1121(60.2)	

## Discussion

This research reveals that the prevalence of inadequate FV intake in an adult population (18-84 years) in northern Angola was 86.7%. This prevalence was higher than reported in South Africa^6^ but very similar to studies conducted in Tanzania^8^ and Uganda^14^. However, it is markedly lower than the prevalence reported in Mozambique, Ethiopia, and Kenya, where authors found insufficient FV intake prevalences of 95.8%, 98.5%, and 94.0%, respectively^7^,^9^,^12^. The low prevalence of adequate fruit and/or vegetable consumption in sub-Saharan Africa has been attributed to variations in the availability of fruits and vegetables in the region, as well as cultural dietary patterns and a growing urbanization rate on the continent, not excluding the low purchasing power of the population^26^. Several studies in sub-Saharan Africa report that women have significantly higher adequate FV consumption compared to men^8^,^12^,^27^ or find no difference between genders^14^,^28^,^29^. In our study, we found precisely the opposite, with a higher proportion of women having inadequate FV intake, corroborating data from a study conducted in South Africa^6^ and another in Kenya^13^.

In our work, low education was associated with inadequate FV intake, in line with various studies conducted in sub-Saharan Africa^28^,^30^–^33^. Greater knowledge about the benefits of FV intake may explain this difference and is often cited in the literature as the main justification^34^–^37^. In this study, we found no difference in the average ages of individuals with adequate FV consumption compared to those consuming less than 5 servings/day of FVs. However, we observed that individuals under 20 years and over 50 years consumed fewer FVs than other age groups. Except for the study conducted by Msambichaka et al. in Tanzania^8^, which states that older age groups are more likely to have adequate FV intake, the majority of studies did not find this association^14^,^28^,^29^. Contrary to what other authors have reported, our study found no association between FV intake and family income and marital status. Studies conducted in Ethiopia, Uganda, and Tanzania are consistent, showing that married or cohabiting individuals, as well as those with higher family income, are more likely to have adequate FV intake^8^,^12^,^38^–^40^.

Healthy diets, especially those including FV consumption, are more commonly observed among people who are married or live with others, especially among men^41^. Conversely, although not always, families with higher monthly income are more likely to include FVs in their diets^35^,^42^,^43^. In a survey conducted in the Imbondeiro neighborhood in Luanda, Angola, among low-income populations, the 588 participating adults reported that regarding dietary habits, 94.3% consume some form of carbohydrates (rice, pasta, potatoes, and funje) every day, 37% consume processed meats 3 or more times per week, 34.4% consume sweets 3 or more times per week, and 25.6% daily, with no mention of FV consumption^44^. In this study, we found no association between FV intake and unhealthy lifestyles such as smoking and alcohol consumption. Two studies conducted in sub-Saharan Africa evaluated this association; in Msambichaka et al.'s study, individuals who consumed alcohol daily were more likely to have adequate FV consumption, while the same did not apply to smokers^8^. Conversely, Peltzer et al. report precisely the opposite, where smokers were more likely to have inadequate FV intake, with no association between alcohol users and FV intake^6^. Lastly, as reported by other authors, our study found no association between FV intake and clinical conditions such as hypertension, hyperglycemia, and hypercholesterolemia^6^,^45^.

## Limitations

To our knowledge, this is the first study to address FV consumption patterns in Angola based on a large representative sample of the population, but some limitations should be discussed. In this study, participants were asked to record their own food intake, which may result in inaccuracies due to memory, social influences, and potential assessment errors. Using individual reports to quantify the diet presents challenges in diet and nutritional epidemiology research, including potential underestimation or overestimation of consumption, which may represent a methodological limitation in this work.

## Conclusion

In this study, the prevalence of inadequate FV consumption was high, and the majority of the studied population did not consume an equal or higher number of 2 servings of fruits/day or 3 servings of vegetables/day. Women, low education levels, and individuals aged 20-29 were associated with inadequate fruit and vegetable intake. There was no association between fruit and vegetable consumption and cardiovascular risk factors, but a prevalence of fruit and vegetable consumption well below current recommendations was identified. The identified risk factors can serve as a local strategy to increase FV consumption. Increasing the consumption of fruits and vegetables should be a priority for our country, as it constitutes a significant factor in the development of non-communicable chronic diseases.
